# Endocrine Resistance in Breast Cancer: The Role of Estrogen Receptor Stability

**DOI:** 10.3390/cells9092077

**Published:** 2020-09-11

**Authors:** Sarah A. Jeffreys, Branka Powter, Bavanthi Balakrishnar, Kelly Mok, Patsy Soon, André Franken, Hans Neubauer, Paul de Souza, Therese M. Becker

**Affiliations:** 1 Centre for Circulating Tumour Cells Diagnostics & Research, Ingham Institute of Applied Medical Research, Liverpool NSW 2170, Australia; branka.powter@inghaminstitute.org.au (B.P.); patsy.soon@health.nsw.gov.au (P.S.); andrechristian.franken@med.uni-duesseldorf.de (A.F.); paul.desouza@health.nsw.gov.au (P.d.S.); therese.becker@inghaminstitute.org.au (T.M.B.); 2 School of Medicine, Western Sydney University, Campbelltown NSW 2560, Australia; 3 Department of Medical Oncology, Liverpool Hospital, Liverpool NSW 2170, Australia; bavanthi.balakrishnar@health.nsw.gov.au (B.B.); kelly.mok@health.nsw.gov.au (K.M.); 4 South Western Sydney Clinical School, University of New South Wales, Liverpool Hospital, Liverpool NSW 2170, Australia; 5 Department of Surgery, Bankstown Hospital, Bankstown NSW 2200, Australia; 6 Department of Obstetrics and Gynaecology, University Hospital and Medical Faculty of the Heinrich-Heine University Düsseldorf, 40225 Düsseldorf, Germany; hans.neubauer@med.uni-duesseldorf.de; 7 School of Medicine, University of Wollongong, Wollongong NSW 2522, Australia

**Keywords:** breast cancer, *ESR1*, proteasome, posttranslational modifications, endocrine therapy

## Abstract

Therapy of hormone receptor positive breast cancer (BCa) generally targets estrogen receptor (ER) function and signaling by reducing estrogen production or by blocking its interaction with the ER. Despite good long-term responses, resistance to treatment remains a significant issue, with approximately 40% of BCa patients developing resistance to ET. Mutations in the gene encoding ERα, *ESR1*, have been identified in BCa patients and are implicated as drivers of resistance and disease recurrence. Understanding the molecular consequences of these mutations on ER protein levels and its activity, which is tightly regulated, is vital. ER activity is in part controlled via its short protein half-life and therefore changes to its stability, either through mutations or alterations in pathways involved in protein stability, may play a role in therapy resistance. Understanding these connections and how *ESR1* alterations could affect protein stability may identify novel biomarkers of resistance. This review explores the current reported data regarding posttranslational modifications (PTMs) of the ER and the potential impact of known resistance associated *ESR1* mutations on ER regulation by affecting these PTMs in the context of ET resistance.

## 1. Introduction

The estrogen receptor (ER) is the main downstream effector of its ligand estrogen and has functions connected to the menstrual cycle, pregnancy, and lactation in females and in maintaining cardiovascular, nervous, musculoskeletal, and immune system functioning [1]. There are two subtypes of ER, namely ERα and ERβ, encoded by *ESR1* and ESR2, respectively. These genes are located on separate chromosomes, *ESR1* at position 6q24-27 and ESR2 at 14q22-24 [2,3,4]. Whilst highly similar, their ligand binding domains (LBD) differ, enabling specific physiological functions [1,5,6].

**Figure 1:**
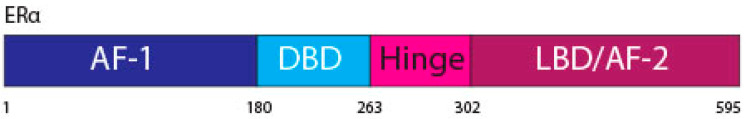
ERα functional domains. The ERα functional domains include; Activation Function 1 (AF-1) (purple), DNA Binding Domain (DBD) (blue), hinge (pink), Ligand Binding Domain (LBD) and AF-2 (plum).

The ERα, a 66 kDa protein, is comprised of four domains, namely activation function 1 (AF-1), DNA binding domain (DBD), the hinge region, and the LBD also known as AF-2 [1,7] (Figure 1).

**Figure 2:**
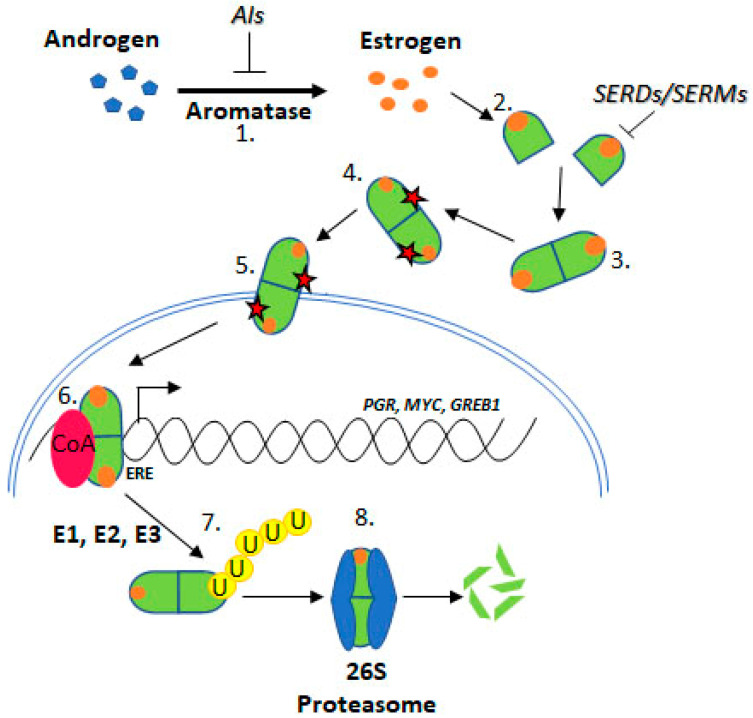
Estrogen Receptor Signalling and ER Targeting Treatment. Aromatase converts androgens to estrogens, Estrogen receptor binds estrogen, Dimerisation, Post translational modification, Nuclear localisation, Coactivator binding and target gene (PGR, c-Myc, GREB1) transcription, Addition of ubiquitin by E1, E2 & E3 ligases, Degradation by the 26S proteasome. Aromatase Inhibitors (AIs) inhibit the enzyme aromatase, Selective Estrogen Receptor Degraders (SERDs) and Selective Estrogen Receptor Modifiers (SERMs) prevent estrogen binding.

Upon estrogen binding, ER forms homodimers (ERα/ERα or ERβ/ERβ) and heterodimers (ERα/ERβ) [8,9]. This review focuses on *ESR1* encoding ERα, referred to as ER henceforth. Following dimerisation, ER translocates to the nucleus and regulates transcription through estrogen response element (ERE) binding within target gene promoters (Figure 2, “genomic function”).

Alternatively, ER may crosstalk with the PI3K-AKT-mTOR or MAPK pathways, in a “non-genomic” manner (see below), in both scenarios promoting cell proliferation and suppressing apoptosis [10].

Approximately 75% of BCa express ER, which promotes oncogenesis. As such, the ER is a common target for BCa treatment using endocrine therapies (ETs) including tamoxifen, fulvestrant, and aromatase inhibitors (AIs) [11] (Figure 2). Tamoxifen is known as a selective ER modifier (SERM) and fulvestrant as a selective ER degrader (SERD) [12]. SERMs competitively bind ER forming an inactive complex, by preventing coactivator interactions [12,13,14,15]. SERDs also competitively bind ER; however, their binding targets the receptor for proteasomal degradation [16,17,18]. AIs are a group of drugs that prevent the synthesis of the estrogen, through inhibition of aromatase [19,20,21]. AIs include letrozole, anastrozole, and exemestane. Whilst these treatments are initially effective for many ER positive BCa patients, resistance remains a significant issue.

Resistance to ET is common, approximately 30% of BCa patients acquire resistance [22,23]. *ESR1* mutations have been identified in ET resistant BCa tumours. Several *ESR1* mutations have been functionally characterised and confer key attributes associated with ET resistance, indicating mechanistic roles in resistance such as estrogen independence, increased transcription of ER target genes like PGR (progesterone receptor), GREB1 (growth regulation by estrogen in breast cancer 1), and MYC (c-myc), and increased proliferation and altered ER conformation [24,25] (Figure 2).

Since a major control of ER activity is through regulation of its half-life, changes to ER stability may influence sensitivity to ET. For example, posttranslational modifications (PTMs) and *ESR1* mutations causing amino acid substitutions at PTM sites may influence ER stability, and ultimately activity. This review explores the link between ER PTMs and turnover, with a focus on ET resistance associated *ESR1* mutations and their effects.

## 2. Estrogen Receptor Signaling

The estrogen/ER complex can result in activation of two distinct types of signaling pathways, the genomic and non-genomic. In the genomic pathway, the ER regulates gene transcription through direct binding of its DBD to promotors containing an ERE, or through interactions with other transcription factors at promoter regions [9,26]. The non-genomic pathway, however, enables rapid signaling through bidirectional crosstalk with PI3K-AKT-mTOR and MAPK pathways. These pathways are frequently upregulated in BCa and enable signaling in an estrogen independent manner [27,28,29]. Several kinases from these pathways phosphorylate the ER at various sites, mediating ER stability, localization, and transactivational capacity, discussed in detail below [30,31]. Further, these pathways may contribute to ET resistance through phosphorylation at key sites, even in the absence of estrogen [32,33,34,35,36,37]. Additionally, crosstalk between ER and PI3K-AKT-mTOR and MAPK pathways is bidirectional. The ER may activate these pathways through interaction with modulator of non-genomic action of estrogen receptor (MNAR) scaffold protein and subsequent SRC activation [38,39]. Ultimately, these pathways promote cell cycle progression through increased expression of cyclin D1, and suppress apoptosis [38,40]. Understanding these interactions is important as the PI3K and MAPK pathways are frequently active in ET resistant BCa and contribute to ET resistance [27,28,29].

**Figure 3:**
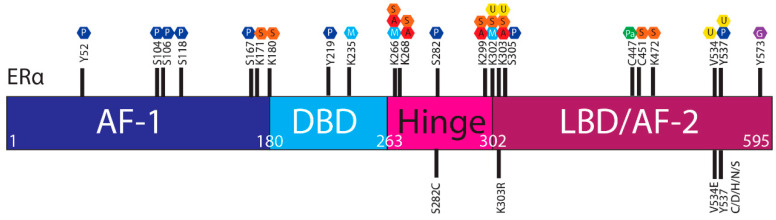
Post translational modifications of ERα. The ERα functional domains include: Activation Function 1 (AF-1) (purple), DNA Binding Domain (DBD) (blue), hinge (pink), Ligand Binding Domain (LBD) and AF-2 (plum). At the top of the schematic are known sites PTMs of ERα; phosphorylation (P, dark blue), SUMOylation (S, orange), methylation (M, light blue), acetylation (A, red), ubiquitylation (U, yellow), pamiltoylation (Pa, green) and gycosylation (G, purple). At the bottom are PTM sites affected by *ESR1* mutations.

The ER is regulated by a range of PTMs including ubiquitylation, SUMOylation, phosphorylation, palmitoylation, acetylation, methylation and glycosylation [41,42,43,44]. These modifications are proposed to regulate the activity, stability, and interactions of ER with other proteins or DNA, and *ESR1* mutations may influence PTMs and hence ER stability and function (Figure 3).

### 2.1. Estrogen Receptor Turnover

Whilst ER is produced through transcription and protein synthesis, changes to ER degradation kinetics is the major factor determining ER levels. In BCa, there is an imbalance between the rate of transcription, synthesis and degradation of the ER leading to increased ER stability and thus activity [45]. The half-life of the ER differs significantly depending on estrogen exposure. Generally, the ER half-life is 3–5 h [46] and estrogen presence can reduce the half-life to just 1 h [47]. However, persistent estrogen exposure causes relative ER stability. In fact, in MCF-7 cells exposed to estrogen for 72 h, ER half-life was increased to 6 h, due to the decreased rate of proteolysis associated with p-S118 [48].

### 2.2. Degradation by the Ubiquitin Proteasome Pathway

Several studies have shown increased ER stability in the presence of the proteasome inhibitor, MG132, indicating that degradation by the ubiquitin–proteasome system (UPS) regulates ER stability [47,49,50,51]. Degradation of the ER occurs predominantly through the UPS, which relies on ER ubiquitylation by ubiquitin activating enzymes (E1) and ubiquitin conjugating enzymes (E2). Conversely, the ER may be protected from degradation through the activity of ubiquitin ligases (E3), which remove ubiquitin from the ER.

Ubiquitin conjugation or removal occurs on lysine residues, and mutations resulting in an exchange, either of such lysines or of amino acids affecting the accessibility of important lysines, may therefore alter the protein’s stability. Additionally, some UPS proteins act as coactivators of nuclear receptors and promote downstream gene transcription [16,44,52,53]. For example, ubiquitin ligase E3A (E6-AP) acts as an ER coactivator and increases gene transcription [16,53].

## 3. Post Translational Modifications

### 3.1. Ubiquitylation

The ER is regulated by both monoubiquitylation and polyubiquitylation and these serve distinct functions. Monoubiquitylation increases the stability and transcriptional activity of the receptor, whilst further addition of ubiquitin, polyubiquitylation, targets the protein for degradation by the UPS [53]. Some proteins have an inhibitory effect on ubiquitylation which leads to increased stability of the ER associated with ET resistance, broadly classified as coactivators, E3 ligases, kinases and scaffold proteins [45].

The K303 site is affected by an *ESR1* mutation, K303R, and is a reported ubiquitination target. K302 and K303 have been implicated in ER stability and its nuclear localisation [52] (Table 1).

The wildtype (WT) K302/K303 ER sites stabilise ligand-free ER and estrogen-mediated ubiquitylation and increase ER transactivational capacity through nuclear translocation. Within the hinge region of the ER, between amino acids K299 and K303, is the nuclear localisation sequence. This sequence is also essential for polyubiquitination and degradation induced by fulvestrant. Mutagenesis of K302 and K303 to alanine (K302R and K303R) result in a disruption to the nuclear localisation sequence [16]. Additionally, lysines central to ubiquitin binding are removed, which causes reduced ER degradation thus, confirming their role in regulating ER stability and antiestrogen resistance [52]. The K302R and K303R mutants are resistant to ubiquitylation by breast cancer type 1 susceptibility protein (BRCA1), hence promoting ER stability [73] and opposing fulvestrant induced degradation [52]. However, some data suggest that BRCA1/BRCA1 associated RING domain 1 ubiquitylates K302/K303 mutant ER at alternative, nearby lysines instead lead to a reduced rate of degradation compared to WT [64].

The Y537 site, although not a lysine, is also important in ubiquitylation and subsequent degradation. In response to estrogen binding, ER associates with the proto-oncogene tyrosine-protein kinase Src (SRC). SRC phosphorylates the ER at various sites, including Y537 [70]. Y537 phosphorylation favours interaction of ER with E6-AP ligase, enhancing ER binding to target promoters [70]. Following ER driven gene transcription, the E6-AP ligase polyubiquitylates the ER targeting it for degradation [70]. Mutagenesis to Y537F results in reduced interaction with the E6-AP ligase, reduced transactivation of ER target genes, including GREB1 and pS2, and increased stability [70]

### 3.2. SUMOylation

Small ubiquitin-related modifier 1 (SUMO-1), shares structural similarities with ubiquitin and has a similar mechanistic pathway, involving activating, conjugating and ligase enzymes. Despite this, SUMO-1 has distinct functions to ubiquitin. The SUMO pathway regulates transcription, nuclear transport, cell growth, proliferation, apoptosis, and protein stability and activity, which are important cellular processes in carcinogenesis [57]. The SUMOylation pathway relies on the actions of activating (SUMO Activating Enzyme), conjugating (ubiquitin-conjugating enzyme E2) and ligase enzymes (protein inhibitor of activated STAT) [57]. Whilst the ER lacks the classical ΨKXE SUMOylation site, SUMOylation at lysines K171, K180, K266, K268, K299, K302, K303, and K472, within the hinge region, has been reported [56,57] (Table 1).

ER SUMOylation regulates the transcriptional activity of the receptor in response to estrogen and tamoxifen. Treatment with estrogen and with tamoxifen enhances SUMOylation required for transcriptional activity. In a study by Sentis et al. mutation of ER lysine to arginine mutations at several positions (K266, K268, K299, K302, and K303) significantly reduced SUMOylation and gene transactivation. However, in that study, several mutations were investigated simultaneously, which explains why the effects of individual lysine to arginine mutations were not assessed [57].

SUMOylation is required for fulvestrant induced transcriptional repression, and is associated with recruitment of corepressors and reduced chromatin accessibility [56,69]. In the presence of fulvestrant, SUMOylation may only allow transient DNA binding of the receptor, reducing overall transcription [69]. However, another study suggested that fulvestrant induces SUMOylation at K171, K180, K99, K472 sites. Furthermore, mutagenesis of L539A or L540A reduces this SUMOylation and hence promotes gene transcription in the presence of antiestrogens [56]. This notion is supported by the fact that the ET-resistance ER mutation V534E, which prevents SUMOylation, also enhances ER dependent gene transcription in the presence of fulvestrant [69]. Therefore, SUMOylation may be required for the effect of antiestrogens, and can be influenced by mutations in the LBD of the ER.

### 3.3. Phosphorylation

Phosphorylation is a common PTM regulating the activity of the ER, with various kinases phosphorylating approximately 20 ER sites. ER phosphorylation affects ligand binding, DNA binding, dimerisation, transcription, coactivator binding, protein stability, subcellular localisation, interactions with other PTM effectors, and cell growth and invasion [74]. Here, we focus on those involved in ER stability and ET resistance or occurring at sites of *ESR1* mutations: Y52, S104, S106, S118, S167, Y219, S282, K303, S305, and Y537 (Table 1). Many studies have investigated the effects of ER phosphorylation, using mutagenesis to change amino acids at serine or tyrosine phosphorylation sites to either prevent (mutagenesis to alanine or phenylalanine, respectively) or mimic (serine to glutamic acid) phosphorylation. The data support the premise that S104, S106, and S118 phosphorylation is essential for ER transactivational activity. Moreover, alanine substitution inhibits, whilst a change with glutamic acid enhances, ER target gene transcription, compared to WT [31]. At ER S167 and S118 sites, phosphorylation loss results in increased growth and migration/invasive capacity, distinct gene expression patterns, and a reduction in apoptosis, in MCF-7 cells [75]. S167 phosphorylation, however, stabilises ER. In the presence of the protein synthesis inhibitor, cycloheximide, in 293T cells ectopically expressing ER S167A, its levels were reduced compared to ectopic WT ER. MG132 restored ER levels in S167A mutant expressing cells, demonstrating regulation of stability by the UPS [30].

ER S118A and S118E mutants on the other hand were more stable than WT ER, and S118E displayed higher transcriptional activation capacity, whilst S118A reduced gene transcription, compared to WT. This suggests that the S118 site has mechanistically distinct roles in regulating ER degradation and ER transcriptional activity [76]. Following estrogen exposure and subsequent gene transcription, a portion of the total receptor pool are ubiquitylated leading to degradation; this response is rapid, occurring within the first 24 h [48]. Meanwhile, another portion of ER is phosphorylated at S118, effectively preventing ubiquitination and thus degradation. Thus, over time, 24–72 h post estrogen addition, the rate of proteolysis decreases [48]. Thus, the S118 site of ER maybe central to controlling the tight balance of activity and protein turnover.

Phosphorylation at Y52 and Y219 ER sites by c-Abl individually enhances gene transcription and ER stability [54]. Y219 phosphorylation also promotes DNA binding and dimerisation [54]. Mutagenesis of these sites (Y52F and Y219F) causes a reduction in cell number and invasive capacity [54]. Interestingly, c-Abl also inhibits the proteasome, through phosphorylation of a subunit known as PSMA7, and thus may reduce ER degradation [54]. c-Abl extends the half-life of the ER from 6 h (c-Abl inhibited) to 12 h (c-Abl active), with no observable change in the mRNA levels of ER [54]. Likewise, inhibition of the 26S proteasome by MG132 extends the half-life of ER, even after c-Abl inhibition [54].

Phosphorylation also regulates tamoxifen response. The effect of tamoxifen on the ER is context dependent in that it can either block ER (antagonist) in breast tissue and it can promote ER activity (agonist) in other tissues through activation of AF-1 [77]. For instance, phosphorylation of S104, S106 and S118 promotes tamoxifen agonist activity in the breast [31]. COS-1 cells expressing S106E, mimicking phosphorylation, had greatest activity followed by S104E and S118E, in the presence of tamoxifen [31]. Meanwhile, phosphorylation of the S282 site is thought to contribute to tamoxifen sensitivity. Patients treated with tamoxifen with phosphorylated S282 ER had longer progression free survival (PFS) and overall survival (OS) than patients lacking phosphorylation at this site [37]. The phosphorylation at S282 also plays an important role in ER stability. COS-1 cells ectopically expressing ER S282A mutant displayed reduced mutant ER levels at 24 h, but not as early as 3 h, of estrogen exposure, compared to WT ER expressing cells [61].

The S305 ER site is phosphorylated by protein kinase A (PKA) and p21-activated kinase 1 (PAK1), and, in some cases, by Aurora Kinase A and AKT [33,67,78,79]. Phosphorylation of S305 in K303R ER mutants enhances interaction with insulin-like growth factor 1 (IGF-1) and is associated with constitutive phosphorylation of IGF-1 and insulin receptor substrate (IRS); promoting cell growth [33]. This cell growth is reduced by inhibiting IGF-1 receptor (IGF-1R) with the tyrosine kinase inhibitor, Tyrphostin AGTreatment with both the AI substrate androstenedione and IGF-1 resulted in greater growth of K303R mutant cells, than with either of these ligands independently [33]. These data provides further evidence of the crosstalk between K303R ER and the IGF-1/IRS/AKT pathway and suggest that phosphorylation of S305 plays critical roles in ER protein interactions and in AI resistance [33].

ER S305 phosphorylation by PKA and PAK1 is associated with tamoxifen resistance and is a potential predictive marker of tamoxifen treatment response [34,35,80]. S305 phosphorylation causes tamoxifen bound ER to remain in its active conformation, thereby promoting resistance [35]. Indeed, BCa patients with tumours that had nuclear PAK1 expression and phosphorylated ER S305, had significantly reduced responses to tamoxifen; nuclear expression of phosphorylated ER S305 was observed in 36% of patients and showed a trend towards reduced tamoxifen response [80]. However as less than 10% of tumours had both phosphorylated ER S305 and nuclear PAK1 expression, this suggests some redundancy and other kinases, such as PKA, may also be involved in the phosphorylation of ER S305 [80]. It was proposed that PKA phosphorylates tamoxifen bound ER at the S305 site, and this alters its conformation such that the coactivator SRC can facilitate transcription factor complex assembly and localisation to target promoters, to enable gene transcription in the presence of tamoxifen [67]. Indeed, activation of PKA through the down regulation of a negative regulator of PKA, known as PKA-Riα, was predictive of tamoxifen resistance and resulted in a growth stimulatory effect in the presence of tamoxifen, interestingly not observed when treated with fulvestrant [35]. These studies suggest that phosphorylation of ER S305, by either PKA or PAK1, is linked with resistance to tamoxifen and AIs.

The Y537 site is phosphorylated by SRC, following ER ligand binding. As outlined above, p-Y537 attracts E6-AP ubiquitin ligase, promoting transactivational activity and ultimately degradation, linking these PTMs functionally [70]. ER Y537 phosphorylation promotes cytoplasmic localisation, however, is associated with increased proliferation [81]. While it remains counterintuitive that cytoplasmic p-Y537 ER increases proliferation, it was speculated that proliferation occurs as a result of growth signaling pathway interactions which promote cell cycle progression [81]. Indeed, there is evidence that p-Y537, while less transcriptionally active, cooperates with phosphorylated SRC to drive proliferation via the AKT pathway and downstream cyclin D1 upregulation [81]. In MDA-MB-231 cells ectopically expressing ER Y537F, ER is retained in the nucleus, preventing its extranuclear activities [70]. There, ER Y537F is stabilised likely due to a lack of E6-AP interaction needed for proteasomal degradation within the nucleus [70] These data highlight the importance of Y537 in ER regulation and may help explain its frequency as AI resistance associated ER mutations. Further study is needed to fully elucidate the effects of p-Y537 and the effects of loss of this functional PTM via amino acid substitutions.

One resistance associated change is Y537S, and the substituted serine may be a target of phosphorylation but that has not been investigated to-date. One study, however, observed increased S118 phosphorylation for ER Y537S mutants [82], associated with increased ER stability [48].

It is evident that a large proportion of ER phosphorylation is mediated by kinases belonging to the PI3K-AKT-mTOR and MAPK pathways, mentioned earlier in the context of non-genomic ER signaling. Signaling through these pathways abrogates the necessity for ER binding and contributes to ET resistance. For example, phosphorylation of S167 on K303R mutated ER by AKT, leading to aromatase inhibitor resistance [30,32]. Phosphorylation of ER at S167 by AKT has also been shown to promote ER stability and activate ER mediated transcription in an estrogen independent manner [30,32,83]. Furthermore, ER Y537 phosphorylation by SRC promotes promoter occupancy and gene transcription [70]. These studies demonstrate estrogen independent ER signaling through crosstalk mediated by kinases. Later, potential therapeutics targeting these resistance mechanisms are discussed.

### 3.4. Palmitoylation

Palmitoylation of the ER is important for its stabilisation and localisation. Palmitoylation at C447 drives the association of ER with caveolin-1, which results in membrane localisation and favours non-genomic ER actions (see above) [84]. ER-estrogen interaction promotes removal of palmitoyl groups, the dissociation of caveolin-1, and hence nuclear localisation and ultimately degradation [43,84]. Inhibition of palmitoylation, with 2-bromo-hexadecanoic acid or by C447A mutation, increased ER degradation at all time points investigated, up to 8 h post estrogen treatment [43]. Interestingly, this resulted in reduced ER stabilising S118 phosphorylation, further highlighting relationships between different PTMs at various ER sites [43,48].

### 3.5. Acetylation

Acetylation of the ER occurs within the hinge and LBD region, by the actions of p300/CREB-binding protein at the sites K266, K268, K299, K302, and K303 (Table 1) [60,63]. The effect of acetylation varies dependent on the site. Acetylation at K266 and K268 promotes DNA binding and transactivation capacity [60]. Conversely, acetylation at K302 and K303 represses ER transactivation activity [63]. Phosphorylation at S305 site prevents the acetylation at K303 [33] in turn hypersensitising the ER to estrogen [85]. The ER K303R change, identified in both breast hyperplasia and BCa tissue [66,86] therefore confers estrogen hypersensitivity and prolongs ER stability.

### 3.6. Methylation

Methylation occurs on lysine residues and is catalysed by the histone lysine methyltransferases SET7 and SET and MYND domain containing 2 (SMYND2). SET7 methylates ER at K302 and thereby enhances ER stability, mediates target promoter recruitment and directly competes with ubiquitination, which increases ER stability [59]. ER lysine methylation may also promote interaction with proteins, such as calmodulin, indirectly preventing ubiquitylation mediated by E6-AP also increasing ER stability [59].

The ER hinge region enables the coordination of multiple PTMs in response to the presence/absence of estrogen. For example, K266 methylation and acetylation is dependent on estrogen levels. K266 methylation by either SET7 or SMYND2 represses transactivation activity by preventing ER chromatin recruitment in the estrogen deprived setting [44]. Upon estrogen binding, K266 is demethylated by histone H3K4 demethylase lysine-specific demethylase 1 [44]. Subsequently, p300/CBP acetylates the ER, permitting chromatin recruitment and gene transcription [44]. K235 is also methylated by SMYND2, in the absence of estrogen, acting to repress gene transcription. Crosstalk between methylation and acetylation at this site is mediated by the transcriptional coactivator euchromatin histone methyltransferase 2, which recognises demethylated K235 [87]. This results in recruitment of the ‘PHD finger protein’/’males absent on the first’ complex, which is responsible for the acetylation of the ER and results in transcriptional activity [87].

### 3.7. Glycosylation

Glycosylation of S573, facilitated by a polypeptide known as N-acetylgalactosaminyltransferase 6 (GALNT6), promotes nuclear localisation, transcriptional activity and stability of the ER (Table 1). In T47D and MCF-7 cells, siRNA knockdown of GLANT6, results in reduced ER nuclear localisation and expression of ER target genes (MYC, CCND1 and CTSD) and this was generally irrespective of estrogen presence [41]. Hence, S573 glycosylation by GLANT6 is an important regulator of ER localisation and transcriptional activity [41].

## 4. Clinical Relevance

ER signaling remains central to the treatment of ER positive BCa, and PTMs play a significant role in determining responses to ET, alongside *ESR1*/ER mutations. Table 1, outlines the functions of common ER PTMs, including phosphorylation, acetylation, ubiquitylation and SUMOylation, and known *ESR1*/ER mutations at these sites. Resistance mechanisms to ET are complex, and likely involve changes to number of pathways with ER at a central hub. While ER PTMs and their dysregulation due to mutations would affect the interactions of ER with several signaling pathways, including growth signaling and proteasomal degradation pathways, this presents an opportunity whereby combination therapies with drugs targeting these pathways may help to re-sensitise tumours to ET.

### 4.1. Estrogen Receptor Mutations and Resistance

Several *ESR1* mutations have been identified. The general consensus is that these mutations may cause conformational changes that affect drug binding and/or ER activity. For this review it is important to emphasise that four key mutation sites are also sites of PTMs, namely S282C, V534E, K303R and Y537C/N/S, described below (Table 1).

#### 4.1.1. S282C

The S282C *ESR1* mutation was identified in a single BCa tumour [62]. Functionally, the effect of this mutation has not been tested, but p-S282 has been proposed to inhibit ligand dependent activation of the ER. The S282A ER mutation prevents phosphorylation and increases ligand independent transcriptional activity compared to WT [61]. The S282C mutation detected in a patient’s tumour tissue would likely also prevent phosphorylation and increase ligand independent ER transactivation activity. Due to its rarity, the clinical relevance of this mutant, however, may be minimal.

#### 4.1.2. K303R

The K303R *ESR1* mutation was first identified in breast typical hyperplasia patients, with a frequency of 34% in (20/59) of ET resistant BCa patients [86]. In invasive BCa, K303R was detected in 50% (133/267) of patients and was associated with reduced 10-year recurrence free survival, larger tumour size and lymph node involvement [66]. Functional testing showed that ER K303R is hypersensitive to estrogen, binds to PI3K to promote growth and survival via the PI3K/pathway, and in combination with p-S305, promotes signaling through the IGF-1R/IRS-1/AKT pathway. It is through these pathways that the K303R mutation may promote resistance to tamoxifen and anastrozole [32,33,65]. The effect of K303 is mediated by PTMs and connected to other posttranslational events as the phosphorylation of S305 inhibits acetylation of K303 resulting in ER inhibition [85]. Some of these functional changes may be based on the K303 site being a common PTM site for ubiquitylation, SUMOylation and acetylation, dependent on the context. Thus, a substitution from lysine to arginine at position 303 likely has important implications for ER activity.

#### 4.1.3. Y537C/S/N

There are number of mutations identified at Y537, including Y537C, Y537D, Y537H, Y537N, and Y537S [25,45]. P-Y537 is critical to control ligand binding, DNA binding, dimerisation, transcription, coactivator binding, protein stability, subcellular localisation, interactions with other PTMs, and cell growth and invasion [58]. Y537N and Y537S were detected in 13.89% of biopsies from patients with hormone resistant tumours and accounted for over half of all detected *ESR1* mutations [24]. Y537S confers resistance to fulvestrant treatment, and is associated with reduced PFS, and occurs at a higher frequency following fulvestrant treatment 8.7% (17/195) compared to 1.5% (3/196) in advanced BCa patients [88]. In another study, *ESR1* mutations were detected in CTCs exclusively from patients treated with estrogen deprivation therapy, and of the 13 patients with *ESR1* mutations detected in CTCs, 23% (3/13) had Y537C/N mutations [89]. The outlined importance of PTMs at the Y537 site suggests that *ESR1* Y537 mutations may at least in part cause resistance by affecting ubiquitylation and phosphorylation and the connected molecular outcomes of these PTMs.

### 4.2. Other PTM Sites and Resistance

#### S167 and S118

Several studies have investigated the effect of phosphorylation at S167 and S118 and the resulting sensitivity to tamoxifen. Reports with regard to p-S167 are conflicting with some studies observing no association, some reporting trends of association and others showing correlation, between phosphorylation loss and tamoxifen resistance [90,91,92,93]. The effect p-S118 site is also controversial. In patients with primary tumours expressing p-S118, it was reported that tamoxifen treatment assured longer disease-free survival (DFS) and a trend towards better OS [94]. Conversely, another study demonstrated that patients whose primary tumours were positive for p-S118 had significantly shorter DFS and OS after treatment with tamoxifen [91]. Low phosphorylation levels at S118 was associated with improved DFS and OS [63] and elevated p-S118 levels were associated with relapse following tamoxifen treatment [95]. Interestingly, another study investigated the effect of the S118P variant on endocrine response and found no significant difference in response to either tamoxifen or fulvestrant [96]. Whilst these studies may differ in patient cohorts and methods, it is possible that the effect of the phosphorylation is context dependent and may relate to other PTMs and pathways. A number of studies have observed tamoxifen resistance in association with p-S305 in both in vitro studies and in patient cohorts [97,98].

### 4.3. Treatments

Treatment of metastatic ER positive BCa is challenging with 30 of patients developing resistance to ET [22,23]. Therapy options for these patients can be broadly categorised into one of three groups, based on their targets: (i) those specifically targeting the ER, (ii) those targeting ER signaling pathways, and (iii) those targeting alternative pathways [99]. Treatment can be a monotherapy or a combination of drugs from these different categories, targeting mutated ER, degradation (proteasome inhibitors), or kinase pathways (Table 2).

#### 4.3.1. Targeting ESR1 Mutants

Currently, drugs targeting the ER are categorised as either SERDs or SERMs. Many patients treated with monotherapies of fulvestrant or tamoxifen, for example, develop resistance, in part due to acquired *ESR1* mutations. Novel therapy options for these patients are arising including a novel drug, H3B-5942, which falls into a new ER target category coined “Selective Estrogen Receptor Covalent Antagonists” [101]. H3B-5942 covalently binds to the ER cysteine at position 530, which is located within helix 11 in the ligand binding pocket. ER mutations, at Y537 and D538, are known to stabilise the agonist conformation of the ER [101]. However, upon binding of H3B-5942 an antagonist conformation is adopted, regardless of the presence or absence of the Y537S or D538G *ESR1* mutation [101]. Compared with fulvestrant, H3B-5942 has a greater antiproliferative effect and increased the suppression of ER target genes [101]. Additionally, CDK4/6 inhibitors and mTOR inhibitors, have a synergistic effect when combined with H3B-5942, resulting in greater antiproliferative effects than monotherapy alone [101].

A novel SERD, known as AZD9496, showed anti-tumour activity of AZD9496 in the *ESR1* Y537S mutant in in vitro models, with greater potency than fulvestrant [25,104]. It was also effective in a CTC-174 ER positive BCa patient derived xenograft (PDX) model with the D538G *ESR1* mutation [104]. In a phase I clinical trial for the treatment of ER+/HER- BCa with AZD9496 patients, there was evidence of prolonged disease stabilisation, with good drug tolerability by patients [102]. These data suggest, that AZD9496 may be an effective treatment option for BCa patients with Y537S and D538G mutations. However, in a window-of-opportunity presurgical study, treatment with AZD9496 for two weeks was not superior to fulvestrant at the dose tested [103].

#### 4.3.2. Clinical Proteasomal Inhibition

Proteasome subunits are frequently upregulated and exhibit increased activity in a variety of cancers including BCa [115]. A proteasome inhibitor, bortezomib, has been FDA approved for the treatment of multiple myeloma [116] and several studies are currently investigating its therapeutic potential of in ER positive BCa.

Short-term exposure to bortezomib has been shown to increase ER stability. However, chronic exposure to proteasome inhibition results in reduced transcription of *ESR1* [49,50,51]. Bortezomib is thought to cause chromatin changes of a distal enhancer region of *ESR1* reducing its transcription and consequently ER accumulation [50]. Another study observed a downregulation of ER, Erb-B2 Receptor Tyrosine Kinase 2 (HER2), and epidermal growth factor receptor (EGFR) expression that led to a reduction in PI3K-Akt, mTOR, and MAPK signaling [117].

Bortezomib also inhibits cell growth in ER BCa positive cell lines, by increasing mRNA levels of the cyclin dependent kinase inhibitor (CDKI), p21, causing cell cycle arrest [118]. Interestingly, silencing of the ER prevented the effects of bortezomib, suggesting that ER is required for this p21 regulation [118]. In MCF-7 cells, the combination of bortezomib and tamoxifen inhibited cell growth by 70% [118].

As a proteasome inhibitor, bortezomib has the potential to affect many protein types, and how this contributes to treatment response or toxicity is largely ill defined. The drug was well tolerated in a single agent clinical trial in metastatic BCa patients but had little beneficial effect [119]. Multiple studies have demonstrated that combination therapy of bortezomib and SERDs are effective in the treatment of BCa, including in the ET resistant setting. A randomised phase II trial with the combination of fulvestrant and bortezomib for the treatment of AI resistant metastatic BCa found significantly improved PFS (28.1%), compared to fulvestrant alone (13.6%), despite the median PFS remaining similar. Patients who progressed on fulvestrant alone, were able to change to the combination arm, and in this group PFS was at 18% for a minimum of 24 weeks [120].

Fulvestrant causes the accumulation of newly synthesised ER aggregates in the cytoplasm. In the presence of bortezomib, these aggregates are not degraded and hence build up within the cytoplasm, leading to the initiation of what is described as the “proapoptotic unfolded protein response”, leading to cell death [121]. For any treatment, identification of patient groups that are likely responders is essential; one study identified a proteasome signature which was associated with poorer patient outcomes and demonstrated that MCF10A cells expressing this signature were susceptible to Bortezomib treatment [116].

#### 4.3.3. Targeting Kinases

ER p-S305 is strongly associated with ET resistance. This site is phosphorylated by PAK1, PKA, Aurora Kinase A or AKT [33,78] and overexpression of PAK1 and PKA is associated with poorer patient outcomes in BCa. Additionally, both PAK1 and PKA induced S305 phosphorylation are strongly correlated with reduced time to progression. Patients with p-S305 progressed in nine months, compared with 18 months seen in patients without PAK1/PKA associated S305 phosphorylation [97]. The *ESR1* K303R mutation, which is associated with tamoxifen and AI resistance, increases the efficiency of S305 phosphorylation [32,33,78]. As this site may be phosphorylated by three different kinases, targeted therapy could be challenging, but may also provide the opportunity to target based on context. Several studies have demonstrated that inhibiting S305 phosphorylation, in in vitro and in xenograft models, may re-sensitise ET-resistant tumour cells to ET and cause increased apoptosis.

A clinical trial has investigated the combination of alisertib with fulvestrant as a therapeutic option for BCa patients [108]. Alisertib is a drug which targets Aurora Kinase A, which is another kinase associated with ET resistance and which phosphorylates the ER at S305 [79]. In a phase I study, the combination of alisertib and fulvestrant had a positive anti-tumour activity, in ET resistant patients, which had been previously treated with tamoxifen and AIs. A six-month clinical benefit rate was achieved in 77.8% of patients [108].

FRAX1036 is PAK1 inhibitor and has been combined with Alisertib with promising results in a range of BCa cell lines, and in BT474 xenograft models. The combination acts synergistically, to inhibit growth and ER signaling and reduces cell survival and its greatest efficacy was observed in hormone receptor positive/HER2 positive BCa cell lines [109]. Furthermore, it was observed that this combination inhibits phosphorylation of ER S305 and S118 in BT447 and T47D cell lines, and prevents PAK1 and ER signaling in BT474 xenograft models [109].

PAK1 activation can be blocked by the histone deacetylase inhibitor, FK228, which suppresses estrogen dependent growth in MCF-7 cells, and blocks growth in tamoxifen sensitive and resistant xenograft models [105]. Other histone deacetylase inhibitors have shown promising results in treating ET resistant BCa including a phase II study where 40% of patients resistant to AIs achieved tumour regression or disease stabilisation in response to the combined treatment with histone deacetylase inhibitor, vorinostat, and tamoxifen [106].

As mentioned previously, AKT can phosphorylate S305, in the presence of K303R. Given the significant crosstalk between the PI3K/AKT pathway and the ER, it could be a potential therapeutic target. In a phase II study, an AKT inhibitor, capivasertib, was tested in combination with fulvestrant in patients who had progressed on AIs. Patients who received capivasertib plus fulvestrant had a median PFS of 10.3 months compared to 4.8 months in patients who received a placebo plus fulvestrant, warranting further investigation in a phase III trial [107].

#### 4.3.4. Targeting Coactivators

Another approach to the treatment of ET resistant BCa is to identify druggable targets in the resistance pathways. ER Y537S and D538G expressing BCa cells are reliant on SRC activity. Unlike WT, these ER mutants can bind the coactivator SRC, even in the absence of estrogen [96,122]. As such, SRC inhibition may be an effective therapeutic option. The combined treatment with a SRC inhibitor, SI-1, and the SERD AZD9496 resulted in a significant reduction in tumour volume compared with monotherapy and controls, in a PDX ER Y537S expressing mouse model [122].

Several studies have also investigated SRC inhibitor potential in ET resistant BCa, particularly combination with AIs or SERDs. The combination of the AI, letrozole, and the SRC inhibitor dasatinib resulted in a clinical benefit rate (the percentage of patients with complete response, partial response or stable disease ≥6 months) of 71% for patients treated with the combination, 66% for those treated with letrozole alone and 23% for those that progressed on letrozole alone and crossed over to the dasatinib/letrozole combination [110]. It was proposed that the addition of dasatinib may delay resistance to letrozole [110].

Saracatinib (AZD0530), another SRC inhibitor, may also be effective in combination with ET, such as fulvestrant or AIs. The combination of saracatinib with fulvestrant reduced proliferation and to a greater extent than either drug alone in both MCF-7Arom5 cells, which express aromatase, and xenograft mouse models, suggesting a synergistic effect [111]. The combination of saracatinib and anastrazole achieved greater cell cycle arrest than either drug alone, and in xenograft models had greater antitumour activity, supporting further clinical investigation [112].

## 5. Conclusions

The ER and its signaling remain central to BCa treatment and ET resistance poses significant challenges. Since PTMs significantly modify ER functions, including protein interactions, subcellular localisation, estrogen sensitivity, transactivation capacity, and stability, amino acid substitution mutations altering PTM sites may dramatically change ER effects and responses to ET. The S282, K303, and Y537 sites play key roles in ER regulation and are sites of *ESR1* mutations associated with ET resistance. Mutations at these sites will prevent ubiquitination (K303 and Y537), SUMOylation (K303), phosphorylation directly (S282, Y537, and indirectly at S305), and acetylation (K303). Here, we reviewed ample evidence that these changes affect ER function and half-life and ultimately change the response to ET. This review has highlighted the factors contributing to ET resistance and promising therapeutic options for the treatment of ET resistant BCa, particularly in patients with *ESR1* mutations.

## Figures and Tables

**Table 1:** ER Posttranscriptional Modification Sites and Effects of Mutations.

Site	Posttranslational Modification (Mediator)	Effect	*ESR1* Mutations ^#^	ET Resistant	Frequency	Functionally Tested
Y52	Phosphorylation (c-Abl)	Increased gene transactivation, increased DNA binding and dimerisation, rapid cell growth and increased invasive capacity [54]	None	N/A	N/A	N/A
S104	Phosphorylation (Cyclin A/CDK2, MAPK ERK1/2, GSK3β)	Increased gene transactivation, tamoxifen sensitivity, ER stability in absence of E2 [31,55]	None	N/A	N/A	N/A
S106	Phosphorylation (MAPK ERK1/2, GSK3β)	Increased gene transactivation, tamoxifen sensitivity, ER stability in absence of E2 [31,55]	None	N/A	N/A	N/A
S118	Phosphorylation (CDK7, ERK1/2, IKKα, GSK3β, ILK, EGFR, IGF1R, DNA-PK, RET)	Increased stability [48]	None	N/A	N/A	N/A
S167	Phosphorylation (p90, RSK1, S6 K1, Akt, IKKƐ, CK2)	Inhibits proteasomal degradation [30]	None	N/A	N/A	N/A
K171	SUMOylation (SUMO3)	Repressed gene transactivation, antiestrogen sensitivity [56]	None	N/A	N/A	N/A
K180	SUMOylation (SUMO3)	Repressed gene transactivation, antiestrogen sensitivity [56]	None	N/A	N/A	N/A
Y219	Phosphorylation (c-Abl)	Enhanced gene transactivation, increased DNA binding and dimerisation, rapid cell growth and increased invasive capacity [54]	None	N/A	N/A	N/A
K235	Methylation (SMYND2)	Repressed gene transactivation [44]	None	N/A	N/A	N/A
K266	SUMOylation (SUMO-1)	Repressed gene transactivation [57]	None	N/A	N/A	N/A
	Acetylation (p300)	Promotes DNA binding and transactivation capacity [58]				
	Methylation (SET7, SMYND2)	Repressed gene transactivation [44]				
K268	SUMOylation (SUMO-1)	Repressed gene transactivation [59]	None	N/A	N/A	N/A
	Acetylation (p300)	Enhances DNA binding and gene transactivation [60]				
S282	Phosphorylation (CK2)	Tamoxifen sensitivity, suppression of gene transactivation, ER stability [37,61]	S282C	Unknown	0.3% (1/292) [62]	N/A
K299	SUMOylation (SUMO1, SUMO2/3)	Repressed gene transactivation, antiestrogen sensitivity [56,57]	None	N/A	N/A	N/A
	Acetylation (p300)	Not major target of acetylation, hinge lysines are preferentially acetylated [60,63]				
K302	Ubiquitylation (BRCA1/BARD1)	ER degradation, induced by estrogen or fulvestrant [52,64]	None	N/A	N/A	N/A
	SUMOylation (SUMO-1)	Repressed gene transactivation [59]				
	Methylation (SET7/9, SMYD2)	ER stability, recruitment to promoter [59]				
K303	Ubiquitylation (BRCA1/BARD1?)	ER degradation, induced by estrogen or fulvestrant [52,64]	K303R	Yes, resistant to AIs and tamoxifen [33,65]	49.81% (133/267) of invasive BCa [66]	In combination with S305, promotes crosstalk with growth factor pathways, and confers resistance to AIs and tamoxifen [32,33,65]
	SUMOylation (SUMO-1)	Enhances estrogen induced DNA binding and transcription [52] Repressed gene transactivation [57]				
	Acetylation (p300)	Represses ER transactivation activity [63]				
S305	Phosphorylation (PAK1, PKA)	Tamoxifen resistance In the presence of the K303R mutation, enhances crosstalk with IGF-1/IRS/Akt pathway and aromatase inhibitor resistance [33,34,35,61,67]	None	N/A	N/A	N/A
C447	Palmitoylation (DHHC-7, DHHC-21)	Membrane localisation [68]	None	N/A	N/A	N/A
C451	SUMOylation (SUMO3)	Sensitivity to fulvestrant and tamoxifen, through gene repression [69]	None	N/A	N/A	N/A
K472	SUMOylation (SUMO3)	Repressed gene transactivation, antiestrogen sensitivity [56]	None	N/A	N/A	N/A
V534	Ubiquitylation (E6-AP ligase)	ER degradation, induced by estrogen Subcellular localisation, gene transactivation and the degradation of the ER [70]	V534E	Unlikely	1/616 (0.16%) metastatic BCa [25]	No effect, neither constitutively active nor inactivating [25]
Y537	Ubiquitylation (E6-AP ligase)	ER degradation, induced by estrogen Subcellular localisation, gene transactivation and the degradation of the ER [70]	Y537C	Yes	6/616 (0.97%) metastatic BCa [25]	Ligand independent, somewhat resistant to tamoxifen, fulvestrant and estrogen deprivation [25,71]
	Phosphorylation (SRC)	Regulation of subcellular localisation, transcriptional activity and degradation of the ER [25,45]	Y537D	Unknown	1/616 (0.16%) metastatic BCa [25]	Estrogen independent, increased activation of progesterone receptor [25]
			Y537H	Unknown	1/56 (1.79%) cfDNA [72]	N/A
			Y537N	Yes	5/616 (0.81%) metastatic BCa [25]	Ligand independent, relatively resistant to tamoxifen, fulvestrant and estrogen deprivation [25,71]
			Y537S	Yes	13/616 (2.11%) metastatic BCa [25]	Constitutively active, adopts conformation that enhances coactivator binding, requires much higher concentrations of fulvestrant for inhibition of ER activities than WT [25]
S573	Glycosylation (GALNT6)	Nuclear localisation, gene transactivation [41]	None	N/A	N/A	N/A

# reported mutations in breast cancer.

**Table 2:** Current and Potential Therapeutic Targets in Breast Cancer.

Treatment	Target	Experimental/Clinical Trial/Current Treatment	Outcome/Conclusion
Aromatase Inhibitors (Anastrozole, Exemestane, Letrozole)	Aromatase	Current	The proliferation marker, Ki67, was significantly suppressed by anastrozole (76%) after 2 weeks of treatment compared to tamoxifen (62%) and the combination (64%) [12]
Tamoxifen	ER (SERD)	Current	5-yr adjuvant tamoxifen use results 47% reduction in recurrence [12]
Fulvestrant	ER (SERM	Current	Greater suppression of ER, PgR and Ki-67 was observed in the higher dose fulvestrant [12]
Cyclin-dependent kinase 4/6 inhibitors (CDKIs)	Cyclin-dependent kinase 4/6	Current	CDKI treatment in combination with ET extends PFS by a median of 8.8 months [100]
H3B-5942	ER (SERCA)	Experimental	Greater antiproliferative effect than fulvestrant. Has synergistic effect when combined with CDK4/6 and mTOR inhibitors [101]
AZD9496	ER	Trial	Disease stabilisation [25,102,103,104]
Bortezomib	Proteasome inhibitor	Experimental	Inhibit cell growth of both ER+ and ER- cells [49,50,51]
FK228	Histone deacetylase inhibitor	Trial	Combined with vorinostat and tamoxifen results in tumour regression [105,106]
Vorinostat	Histone deacetylase inhibitor	Trial	Show tumour regression with FK228 [106]
Capivasertib	AKT inhibitor	Trial	Patients who received capivasertib plus fulvestrant had a median PFS of 10.3 months compared to 4.8 months in patients who received a placebo plus fulvestrant, warranting further investigation, in a phase III trial [107]
Alisertib	Aurora Kinase A inhibitor	Trial	In combination with fulvestrant demonstrated anti-tumour activity [108]
FRAX1036	PAK1 inhibitor	Experimental	Acts synergistically with alisertib with greater efficacy [109]
Dasatinib	SRC and AbI inhibitor	Experimental	In combination with letrozole was promising in 71% of patients [110]
Saracatinib (AZD0530)	SRC inhibitor	Experimental	In combination with fulvestrant had a greater effect at reducing proliferation than alone [111,112]
Bosutinib	SRC inhibitor	Experimental	Had an unfavourable risk-benefit ratio [113,114]
